# Access to Health Care and Use of Health Care Services Among Males in Africa: Protocol for a Scoping Review

**DOI:** 10.2196/52351

**Published:** 2025-01-31

**Authors:** Nkoleleng Johannah Mashilo, Kelechi Elizabeth Oladimeji, Siphamandla Gumede, Samanta Tresha Lalla-Edward

**Affiliations:** 1 Ezintsha, Faculty of Health Sciences University of the Witwatersrand Johannesburg South Africa

**Keywords:** health-seeking behavior, health care, access, uptake, services, men, boys, scoping review, Africa, male, health care services, accessibility, use, noncommunicable disease, depression, substance abuse, overdose, physical disability, stress, older men, men’s health, well-being, health literacy, perception, systematic reviews, meta-analysis, electronic database, EHR, electronic health record, narrative synthesis

## Abstract

**Background:**

There is a scarcity of data on males’ health-seeking behavior, as well as their access to and use of health care services, in Africa. According to some studies, men are less likely than women to seek medical help for issues such as communicable and noncommunicable diseases, depression, substance abuse, physical disabilities, and stressful life events. The study of males’ health-seeking behaviors is important, because it allows us to learn about male health, how masculinity encourages underuse of health care services, how this affects males’ overall health and well-being, and how cultural values and backgrounds may impact older men’s health-seeking behaviors.

**Objective:**

The objective of this review is to assess evidence on how males access and use health care services and their health knowledge, attitudes, and perceptions to identify gaps for targeted, context-specific strategies to improve males’ health and outcomes, particularly in Africa.

**Methods:**

The scoping review process will be guided by the methodology frameworks of the Joanna Briggs Institute and Arksey and O’Malley and will follow the Preferred Reporting Items for Systematic reviews and Meta-analysis Protocols Extension for Scoping Reviews guidelines. The following electronic databases will be systematically searched for evidence published between January 2010 and 2023: PubMed, Scopus, Web of Science, African Journals Online, and Google Scholar. Two reviewers will independently screen full texts and chart the data; a third reviewer will be engaged in the event of disagreement between the 2 independent reviewers. The results of this scoping review will be summarized quantitatively through numerical counts and qualitatively through a narrative synthesis.

**Results:**

The electronic database search was conducted between March and April 2023 and redone in April 2024 to include the most recent articles. A total of 114,737 articles were retrieved and 4258 removed as duplicates. After title screening, 337 results remained, and after abstract selection, 140 results remained. As of December 2024, the scoping review was in the full-text screening phase. We plan to complete data extraction, synthesis, and writing of the entire manuscript of the review in January 2025, and then submit it to a journal for peer review and publication in February 2025.

**Conclusions:**

The scoping review results will advance the current knowledge about health-seeking behavior and access to and uptake of health care services among African males. To our knowledge, this scoping review is the first on this topic, and it will identify vital information on the barriers to and facilitators of African males’ health care access and uptake. It will also provide information on successful health care programs for males that may be tailored and adopted across different African contexts.

**Trial Registration:**

OSF Registries https://osf.io/xz6sr

**International Registered Report Identifier (IRRID):**

DERR1-10.2196/52351

## Introduction

The World Health Organization (WHO) defines health as “a state of complete physical, mental and social well-being and not merely the absence of disease or infirmity” [1]. Sustainable Development Goal 3 complements this definition by pinpointing the need for global commitment to “ensure healthy lives and promote well-being for all at all ages” without exclusion [2]. Admirable and essential as these intentions may be, their success is incumbent upon reaching key populations so that the health care requirements of those populations may be fulfilled. Males the world over are one such key population, and they generally underuse the health care opportunities available to them [3-5]. This is concerning, since it is projected that men’s health burden will result in men’s lifespans being 7 years less than women by 2030. In South Africa, the male health risk and mortality rates are high due to tuberculosis, as well as noncommunicable diseases like diabetes and cardiovascular diseases, all of which are mostly preventable [6]. There are various reasons for men delaying or avoiding seeking health interventions, with some of the most regularly identified ones being a paucity of male-centered interventions, sociodemographic factors, and attitudes toward health services [7-9]. Nonetheless, there is evidence that a shift in males’ attitudes is emerging and that they are becoming increasingly willing to access health care. Consequently, there is a drive to not only provide health care services that cater specifically to men and minimize the barriers to their seeking health care, but also to increase research in this field [8]. Our research will thus add to the body of knowledge that is used to fulfill the aforementioned commitment.

“Men” is a broad term; according to the WHO, there are different terminologies, age ranges, and characteristics used to describe the transition from childhood to adulthood for men. Adolescence has been described as the ages of 10 to 19 years and young adulthood as 10 to 24 years [10]. Guided by these clarifications, the age range in our scoping review included all male-related studies involving individuals aged 13 to 17 years as teenagers and those aged 18 years and older as adults. This age range of minors was included due to the fact that the onset of the teenage years is when young people experience “physiological, psychological, and social changes that lay bare the world of sexual experimentation” [11] and the consequences thereof, which may call for health care. This was deemed significant as it could yield a better understanding of males in this population, specifically with regard to their sexual reproductive health practices. Another population that was deemed significant to our study was transgender males, as their health-seeking behavior is currently underresearched, despite their experience of inordinate health risks and challenges [12].

An examination of health policies of 10 low- and middle-income countries revealed that health care priorities in these countries are largely driven by cost-saving measures and the types of disease that are prevalent. This means that health care priorities can differ significantly across geographical areas. Nonetheless, in most sub-Saharan countries, HIV is prioritized, with lymphatic filariasis, syphilis in pregnant or new mothers, human papilloma virus, tuberculosis, and sexually transmitted diseases being other common conditions [13]. What is striking is that not only are none of these conditions unique to males, but some of them are solely female focused. This is concerning because it conveys the impression that particularly male diseases are not a high priority. For example, the incidence of prostate cancer is escalating in West Africa, and treatment is usually delayed for so long that the health outcomes for men with the condition are dismal. Further, men with prostate cancer symptoms either remain ignorant of the disease or are too poor to seek treatment [14]. Equally dangerous are unhealthy lifestyle choices, like smoking and alcohol abuse, which are more prevalent among men, as well as some chronic diseases like diabetes and cerebrovascular disease, two of the main causes of death in men over the age of 45 years: the South African government has recently acknowledged all of these conditions as worthy of being addressed [4]. It is therefore not unreasonable to conclude that improved health literacy targeted specifically at males could help to mitigate their life-threatening health conditions and choices [15].

Apart from the obvious benefits of disease management, males who are inspired and empowered to take control of their own health can inadvertently improve the lives of their families, as well as society in general. Economically, better health would, in the long run, reduce medical costs that would usually be borne by the family and in some part, the government. Socially, healthy men would more likely be able to lead an active life in which they do not feel compelled to avoid health care to preserve their masculinity, nor fear stigmas associated with, for example, mental health conditions or undergoing antenatal HIV screening with their partners [8,16].

But, within the ambit of our study, we first have to ascertain what strategies have the most reasonable chance of uptake to be able to speculate on the benefits of males engaging in health-seeking practices. What has to be borne in mind is that men’s health-seeking behavior, which in Africa is influenced by a particularly patriarchal ethos, limited resources, and faith in traditional medicines and cultural superstitions [8,17], may not necessarily match the behavior of males in countries that do not share similar characteristics.

In essence, this scoping review will seek to describe the variations in health-seeking behaviors among males across the African continent, assess their access to and use of health care services, and identify both challenges and facilitators of health care uptake. Additionally, it will explore males’ knowledge, attitudes, perceptions, and beliefs regarding their health care needs and available services. The overall goal is to assess the current evidence on these factors to guide the development of strategies that enhance males’ health and improve outcomes across the region. To this end, the primary objective of this scoping review is to map evidence on males’ access to and use of health care services and identify gaps for targeted, context-specific strategies to improve males’ health outcomes, particularly in Africa.

## Methods

### Study Design

We will use a scoping review approach to map out existing evidence on males’ health-seeking behavior and access to and uptake of health care services in Africa. This will enable us to identify aspects for tailored intervention to improve males’ health-seeking behavior. In conducting this review, we will apply the methodological approach and review process of the Joanna Briggs Institute (JBI) [18]. The JBI framework enables an extensive and less-biased synthesis of studies by using comprehensive and transparent methods [19]. The JBI framework synthesizes existing knowledge rather than creating new knowledge [20]. This yields decisions that reflect on the feasibility, appropriateness, meaningfulness, and effectiveness of health behavior [19,20] The Preferred Reporting Items for Systematic reviews and Meta-analyses extension for Scoping Reviews (PRISMA-ScR) guidelines [21,22] will be adhered to in reporting this scoping review. The PRISMA-ScR guidelines are congruent with the JBI approach for scoping reviews, which emphasizes the importance of methodological precision for scoping reviews. We believe that the PRISMA-ScR will improve the reporting of our scoping review [22]. According to the 5 main steps [18], we discuss our review process in the following sections.

### Step 1—Identifying the Research Question

The scoping review questions will be formulated according to the population, concept, and context (PCC) approach recommended by the JBI. The PCC framework is suitable when developing objectives and eligibility criteria for scoping reviews [23]. In our study, the PCC inclusion criteria will be useful to guide how the data should be extracted. There is no need for outcomes or interventions of interest to be identified for a scoping review [24] (Table 1).

Based on the PCC framework mentioned above, the research questions we will explore are outlined below:

1. What are the varying health-seeking behaviors among males across the African continent?
2. What are health services for males and how do males access and use health care services in Africa?
3. What knowledge, attitudes, perceptions, and beliefs exist among males about their health care needs and health care services in Africa?
4. What are the challenges and facilitators of health care uptake by males in Africa?

**Table 1:** The population, concept, and context (PCC) elements in this review.

Element	Description
Population	Males only
Concept	Health-seeking behavior; health care service access, use, and uptake
Context	Africa

### Step 2—Identifying Relevant Studies

We will systematically search PubMed, African Journals Online, Web of Science, Scopus, and Google Scholar. The search will include papers with publication years from 2010 to 2023 to better reflect more recent information and current health-seeking behavior among males across the African continent. In addition, government reports available on the websites of departments and ministries of health and associations will be reviewed with a focus on males’ access to health care services, knowledge, perceptions, and attitudes regarding health care services, as well as barriers and facilitators to uptake of health services by males. This will help us to identify reports related to males’ health-seeking behavior in Africa.

The search strategy will include titles, abstracts, and combinations of keywords, as presented in Multimedia Appendix 1 and Multimedia Appendix 2.

### Step 3—Study Selection

After collection of study data from the aforementioned bibliographic databases, 3 reviewers will conduct the review; 2 will perform data selection and database searching and the third will perform quality checking and resolve inclusion and exclusion disagreements between the 2 primary reviewers. The 2 primary reviewers will independently use the inclusion and exclusion criteria to search the titles and abstracts, and they will document reasons for inclusion and exclusion as outlined below. Any discrepancies will be discussed and resolved between these 2 reviewers, but if no conclusion can be reached, the third reviewer will be included in the discussion. Furthermore, the reviewers will independently source full-text versions of articles found to be eligible during the title and abstract screening phase. They will exclude articles without full-text versions available after a conclusive discussion regarding the excluded articles is reached. The searched articles will include male-related studies and include qualitative, quantitative, and mixed methods interventions for males’ health, knowledge, attitudes, and perceptions regarding health services for males, as well as access and challenges to use of health care services. Overall, this scoping review will include material that fulfill the criteria outlined in Tables 1 and 2. The exclusion criteria entail studies not conducted in Africa; not including males’ health care service access and uptake or health-seeking behavior; and those conducted prior to 2010.

**Table 2:** The eligibility criteria.

Criteria for study inclusion	Component details
Setting	Only studies from Africa will be considered for the scoping review.
Language	Only English-language studies will be included.
Date	Only studies from the years 2010 to 2023 will be included.
Publication status	All documented studies will be considered and included in the scoping review. This will include only published peer-reviewed articles.
Method	The study will be designed and reported according to the Preferred Reporting Items for Systematic reviews and Meta-Analyses extension for Scoping Reviews. Population, concept, and context will be used as the search strategy approach. Qualitative, quantitative, and mixed methods studies will be included.

### Step 4—Charting the Data

Data from the identified eligible studies will be collected and charted. Reviewers will record key themes from each eligible article. The themes will include author, journal, publication status, study duration, year, and demographic information (age, ethnicity, marital/relationship status, employment, residential location [rural or urban], income, and education level), geographical information (country and region in which the study was done), research methodology, and findings included in the synthesis (study design, health-seeking behavior, access, uptake, intervention, main results and outcome, limitations, and other important discoveries; Textbox 1). Microsoft Excel, EndNote, and Rayyan will be used in obtaining the data, saving the search, cleaning the data, screening the data, and charting the data for this scoping review.

**Data chart template (to be expanded during actual review).**

**Details of elements included in the synthesis**

- Author
- Journal
- Publication status
- Study duration
- Year

**Demographic information on participants in the synthesis**

- Age
- Residential location (rural or urban)
- Ethnicity
- Marital/relationship status
- Income
- Employment
- Education level

**Geographic information in the synthesis**

- Region where the study was done
- Country

**Research methodology and summary findings in the synthesis**

- Study design
- Health-seeking behavior
- Access
- Uptake
- Intervention
- Main results and outcome
- Limitation
- Other important discoveries

**Other comments, if any**

### Step 5—Collating, Summarizing, and Reporting the Results

A qualitative and quantitative approach will be used to report and summarize the scoping review. The number and type of eligible studies will be reported numerically, and methods, results, health care services, program, and health-seeking behavior information will be described in a narrative form. Tables and charts will be used to map out the screening process and results. Discussion will be guided by developed themes and study objectives and will include definitions of keywords. The risk of bias will not be assessed, as the scoping review is meant to provide an overview of existing literature about health-seeking behavior of African males and not critically analyze or appraise the selected articles; the limitations of the studies will be noted. Secondary analyses will be performed looking at health-seeking behavior and challenges, knowledge, and stigma regarding access to and uptake of health care services among boys and the transgender population. Analyses will also compare males’ health-seeking behavior and service access and use across different regions.

## Results

The electronic database search was conducted between March and April 2023 and was redone in April 2024 to include the most recent publications. A total of 114,737 results were retrieved, and 4258 were identified as duplicates. The database was first title screened, and this yielded a total of 337 articles for further abstract screening, which resulted in 140 articles remaining for full article screening. We are currently finalizing full-article screening for the synthesis. We intend to complete the synthesis and initial manuscript write-up in January 2025, for submission for peer review and publication in February 2025.

## Discussion

### Expected Findings

We expect to substantiate that the burden of disease in males compared to females is high for a plethora of reasons, such as availability of context-specific health care services for males, access by males to health care, and the socioeconomic position of households. Moreover, available evidence has shown that male health-seeking behavior is often delayed, and the uptake of health care services is poor due to barriers such as cultural beliefs, perceptions of masculinity, stigma, gaps in health knowledge, and social and economic factors [25,26]. Based on the limited number of published articles on males’ access to and uptake of health care services, especially in Africa, and observations on trends in the proportions by sex of outpatients (ie, there are more females than males) seeking care in primary health care centers [27-30], there is a need for male health care to be taken into consideration. Facilitators like community-based interventions, male-targeted programs, and peer support have been shown to improve male engagement with health care [26]. To our knowledge, this scoping review is the first on this topic, and it will identify key themes and gaps regarding the barriers to and facilitators of African males’ health care access and uptake. The scoping review will summarize and discuss our findings, including challenges and facilitators of health care service access and uptake and health-seeking behavior among African males. The results may form the basis for future qualitative research on exploring the health-seeking behavior of African males, quantitative research on barriers to and facilitators of access to and uptake of health care services, implementation research on intervention programs to influence African males to access and use health care services voluntarily, or systematic reviews of programs for working African males to access health care and support systems and increase their health care–seeking behavior. The results may also help policy makers to develop programs and interventions specifically tailored for the needs of males through males’ input. The literature synthesis will support the development of evidence-based practices that will address challenges related to males’ access to health care services, enhance their health care knowledge, and promote intervention programs to improve uptake, which may result in a decreased burden of disease and mortality rate among males. We expect our main findings to provide insights into what geography-specific health services are available across the African region, as well as provide the latest evidence on the extent of access and uptake, including contributing factors. The results of this scoping review will be disseminated through scientific conference proceedings and presentations, stakeholder meetings, and by publication in a peer-reviewed journal. During the dissemination of findings, stakeholders will be invited to discuss gaps and challenges affecting males’ health-seeking behavior and the uptake of health care services to develop context-specific strategies that can be implemented to address the identified gaps and challenges. The dissemination report will also include recommendations for future research, review limitations, and discuss the risk of bias.

### Potential Limitations to the Scoping Review

Through this scoping review, we intend to provide a range of information about the health-seeking behavior of males, including their access to and use of health services. However, as we will limit our search to include only published works written in English and implemented in Africa, we are likely to inadvertently exclude relevant programs, thereby skewing the comprehensiveness of the review. Constraints related to the publication type, language, and geographic region will restrict the diversity of the included sources, limiting its applicability across various contexts. Our search strategy did not use the built-in search functionalities of the relevant electronic databases, such as Medical Subject Headings terms for PubMed. Our search strategy might have increased the risk of missing literature. To mitigate this, we constructed a wide range of search terms to ensure a wider reach.

## Appendix

Multimedia Appendix 1 Search permutations.

Multimedia Appendix 2 Search strategy.

## Abbreviations

JBI: Joanna Briggs Institute
PCC: population, concept, and context
PRISMA–ScR: Preferred Reporting Items for Systematic reviews and Meta-Analyses extension for Scoping Reviews
WHO: World Health Organization

## Data Availability

The data sets generated during and/or analyzed during this study are available from the corresponding author on reasonable request.
