# Supplementary material for: Access to Health Care and Use of Health Care Services Among Males in Africa: Protocol for a Scoping Review
Source: JMIR Res Protoc. 2025 Jan 31;14:e52351. doi: 10.2196/52351 (PMC11829170; doi:10.2196/52351)
Supplement: Multimedia Appendix 1 [file resprot_v14i1e52351_app1.docx]

**Appendix 1**

Search term permutations

**a. Men’s healthcare**

i. Men’s healthcare usage

ii. Men’s health care use

iii. Men’s healthcare use

iv. Men’s health care usage

v. Men’s healthcare services

vi. Men's health care services

vii. Men’s healthcare access

viii. Men’s health care access

ix. Men’s healthcare program

x. Men's health care program

xi. Men’s healthcare effectiveness

xii. Men's health care effectiveness

xiii. Men’s healthcare intervention

xiv. Men's health care intervention

xv. Men’s healthcare acceptability

xvi. Men health care acceptability

xvii. Men’s healthcare availability

xviii. Men's health care availability

xix. Men’s healthcare facilities

xx. Men’s health care facilities

xxi. Men’ s healthcare Africa

xxii. Men's health care Africa

xxiii. Men’s health seek

xxiv. Men’s health knowledge seek

xxv. Men’s heath care seeking behaviour

xxvi. Men’s access to health care

xxvii. Men’s treatment seek

xxviii. Men’s health care uptake

xxix. Men’s uptake of health care services

xxx. Men’s health care access

xxxi. Men’s clinic visit

xxxii. Men’s clinic attendance

xxxiii. Men’s health care program

xxxiv. Men’s sexually transmitted infections program

xxxv. Men’s HIV program

xxxvi. Men’s tuberculosis program

xxxvii. Men’s reproductive health program

xxxviii. Men’s communicable disease program

xxxix. Men’s non-communicable disease program

xl. Men’s health condition program

xli. Men’s healthcare service utilization

xlii. Men’s health care service utilization

xliii. Men’s healthcare service utilisation

xliv. Men’s health care service utilisation

xlv. Men’s masculinity

xlvi. Men's cultural practices

xlvii. Men’s traditional practices

xlviii. Men's traditional medicine

xlix. Men's health service strategy

l. Men's health services

li. Men's availability

lii. Men’s intervention

liii. Men’s effectiveness

liv. Men’s friendly services

lv. Men mobile clinic

**b. Males’ healthcare**

i. Males’ healthcare usage

ii. Males’ health care usage

iii. Males’ health care use

iv. Males’ healthcare use

v. Males’ healthcare services

vi. Males’ health care services

vii. Males’ healthcare access

viii. Males’ health care access

ix. Males’ healthcare program

x. Males’ health care program

xi. Males’ healthcare effectiveness

xii. Males’ health care effectiveness

xiii. Males’ healthcare intervention

xiv. Males’ health care intervention

xv. Males’ healthcare acceptability

xvi. Males’ health care acceptability

xvii. Males’ healthcare availability

xviii. Males’ health care availability

xix. Males’ healthcare facilities

xx. Males’ health care facilities

xxi. Males’ healthcare Africa

xxii. Males’ health care Africa

xxiii. Males’ health seek

xxiv. Males’ health knowledge seek

xxv. Males’ heath care seeking behaviour

xxvi. Males’ access to health care

xxvii. Males’ treatment seek

xxviii. Males’ health care uptake

xxix. Males’ uptake of health care services

xxx. Males’ health care access

xxxi. Males’ clinic visit

xxxii. Males’ clinic attendance

xxxiii. Males’ sexually transmitted infections program

xxxiv. Males’ HIV program

xxxv. Males’ tuberculosis program

xxxvi. Males’ reproductive health program

xxxvii. Males’ communicable disease program

xxxviii. Males’ non-communicable disease program

xxxix. Males’ health condition program

xl. Males’ healthcare service utilization

xli. Males’ health care service utilization

xlii. Males’ healthcare service utilisation

xliii. Males’ health care service utilisation

xliv. Males’ masculinity

xlv. Males’ cultural practices

xlvi. Males’ traditional practices

xlvii. Males’ traditional medicine

xlviii. Males’ health service strategy

xlix. Males’ health services

l. Males’ availability

li. Males’ intervention

lii. Males’ effectiveness

liii. Males’ friendly services

liv. Males’ mobile clinic

**c. Transgender healthcare**

i. Transgender healthcare usage

ii. Transgender health care usage

iii. Transgender health care use

iv. Transgender healthcare use

v. Transgender healthcare services

vi. Transgender health care services

vii. Transgender healthcare access

viii. Transgender health care access

ix. Transgender healthcare program

x. Transgender health care program

xi. Transgender healthcare effectiveness

xii. Transgender health care effectiveness

xiii. Transgender healthcare intervention

xiv. Transgender health care intervention

xv. Transgender healthcare acceptability

xvi. Transgender health care acceptability

xvii. Transgender healthcare availability

xviii. Transgender health care availability

xix. Transgender healthcare facilities

xx. Transgender health care facilities

xxi. Transgender healthcare Africa

xxii. Transgender health care Africa

xxiii. Transgender health seek

xxiv. Transgender health knowledge seek

xxv. Transgender heath care seeking behaviour

xxvi. Transgender access to health care

xxvii. Transgender treatment seek

xxviii. Transgender health care uptake

xxix. Transgender uptake of health care services

xxx. Transgender health care access

xxxi. Transgender clinic visit

xxxii. Transgender clinic attendance

xxxiii. Transgender sexually transmitted infections program

xxxiv. Transgender HIV program

xxxv. Transgender tuberculosis program

xxxvi. Transgender reproductive health program

xxxvii. Transgender communicable disease program

xxxviii. Transgender non-communicable disease program

xxxix. Transgender health condition program

xl. Transgender healthcare service utilization

xli. Transgender health care service utilization

xlii. Transgender healthcare service utilisation

xliii. Transgender health care service utilisation

xliv. Transgender masculinity

xlv. Transgender cultural practices

xlvi. Transgender traditional practices

xlvii. Transgender traditional medicine

xlviii. Transgender health service strategy

xlix. Transgender health services

l. Transgender availability

li. Transgender intervention

lii. Transgender effectiveness

liii. Transgender friendly services

liv. Transgender mobile clinics

**d. Boys’ healthcare**

i. Boys’ healthcare usage

ii. Boys’ health care usage

iii. Boys’ health care use

iv. Boys’ healthcare use

v. Boys’ healthcare services

vi. Boys’ health care services

vii. Boys’ health care access

viii. Boys’ healthcare access

ix. Boys’ healthcare program

x. Boys’ health care program

xi. Boys’ healthcare effectiveness

xii. Boys’ health care effectiveness

xiii. Boys’ healthcare intervention

xiv. Boys’ health care intervention

xv. Boys’ healthcare acceptability

xvi. Boys’ health care acceptability

xvii. Boys’ healthcare availability

xviii. Boys’ health care availability

xix. Boys’ health care facilities

xx. Boys’ healthcare facilities

xxi. Boys’ healthcare Africa

xxii. Boys’ health care Africa

xxiii. Boys’ health seek

xxiv. Boys’ health knowledge seek

xxv. Boys’ heath care seeking behaviour

xxvi. Boys’ access to health care

xxvii. Boys’ treatment seek

xxviii. Boys’ health care uptake

xxix. Boys’ uptake of health care services

xxx. Boys’ health care access

xxxi. Boys’ clinic visit

xxxii. Boys’ clinic attendance

xxxiii. Boys’ sexually transmitted infections program

xxxiv. Boys’ HIV program

xxxv. Boys’ tuberculosis program

xxxvi. Boys’ reproductive health program

xxxvii. Boys’ communicable disease program

xxxviii. Boys’ non-communicable disease program

xxxix. Boys’ health condition program

xl. Boys’ healthcare service utilization

xli. Boys’ health care service utilization

xlii. Boys’ healthcare service utilisation

xliii. Boys’ health care service utilisation

xliv. Boys’ masculinity

xlv. Boys’ cultural practices

xlvi. Boys’ traditional practices

xlvii. Boys’ traditional medicine

xlviii. Boys’ health service strategy

xlix. Boys’ health services

l. Boys’ availability

li. Boys’ intervention

lii. Boys’ effectiveness

liii. Boys’ friendly services

liv. Boys’ mobile clinic
