# Supplementary material for: Access to Health Care and Use of Health Care Services Among Males in Africa: Protocol for a Scoping Review
Source: JMIR Res Protoc. 2025 Jan 31;14:e52351. doi: 10.2196/52351 (PMC11829170; doi:10.2196/52351)
Supplement: Multimedia Appendix 2 [file resprot_v14i1e52351_app2.docx]

**Appendix 2**

Search strategy for “**Male access to healthcare and utilization of healthcare services in Africa: Protocol for a scoping review"**

The search strategy will include title, abstract and combination of keywords as follows:

“men” OR “males” OR “transgender“ OR “ boys” OR” health seek” OR” health knowledge seek” OR “health care seeking behaviour” OR “healthcare seeking behaviour” OR “access to health care” OR “access to healthcare” OR” treatment seek” OR “healthcare seek” OR “health care seek” OR “healthcare use” OR “health care use” OR “ healthcare usage” OR “health care usage” OR “ healthcare service utilization” OR “ health care service utilization” OR “ healthcare service utilisation” OR “ health care service utilisation” “heath care service” OR “healthcare service” OR “health care uptake” OR “healthcare uptake” OR “treatment uptake” OR “uptake of health care services” OR “uptake of healthcare services” OR “health care access” OR “healthcare access” OR “clinic visit” OR “clinic attendance” OR “health care program” OR “healthcare program” OR “health care effectiveness” OR “healthcare intervention” OR “health care intervention” OR “healthcare acceptability” OR “health care acceptability” OR “healthcare availability” OR “health care availability” OR “healthcare facilities” OR “health care facilities” OR “ friendly services” OR “ mobile clinic” OR “masculinity” OR “cultural practices” OR “traditional practices” OR “traditional medicine” OR “sexually transmitted infections program” OR “HIV program” OR “tuberculosis program” OR “ reproductive health program” OR “ communicable disease program” OR “non-communicable disease program” OR “ health condition program” OR “health services” AND “ availability” OR “ effectiveness” OR “intervention” OR “health service strategy” OR “ program” AND “Africa “ OR “Algeria” OR “Angola” OR “Benin” OR “Botswana” OR “Burkina Faso” OR “Burundi” OR “Cabo Verde” OR “Cameroon” OR “Central African Republic” OR “Chad” OR “Comoros” OR “Congo” OR “Democratic Republic of the Congo” OR “Republic of the Cote d’Ivoire” OR “Djibouti” OR “Egypt” OR “Equatorial Guinea” OR “Eritrea” OR “Eswatini” OR “Swaziland” OR “Ethiopia” OR “Gabon” OR “Gambia” OR “Ghana” OR “Guinea” OR “Guinea-Bissau” OR “Kenya” OR “Lesotho” OR “Liberia” OR “Libya” OR “Madagascar” OR “Malawi” OR “Mali” OR “Mauritania” OR “Mauritius” OR “Morocco” OR “Mozambique” OR “Namibia” OR “Niger” OR “Nigeria” OR “Rwanda” OR “Sao Tome and Principe” OR “Senegal” OR “Seychelles” OR “Sierra Leone” OR “Somalia” OR “South Africa” OR “South Sudan” OR “Sudan” OR “Tanzania” OR “Togo” OR “Tunisia” OR “Uganda” OR “Zambia” OR “Zimbabwe”)
